# Supplementary material for: HIVprotI: an integrated web based platform for prediction and design of HIV proteins inhibitors
Source: J Cheminform. 2018 Mar 9;10:12. doi: 10.1186/s13321-018-0266-y (PMC5845081; doi:10.1186/s13321-018-0266-y)
Supplement: Supplementary file 2 — Additional file 2. Source code of HIVProtI web server. [file 13321_2018_266_MOESM2_ESM.zip › HIVprotI_Source-code/hivprotI/weka-3-6-6/doc/weka/gui/treevisualizer/NamedColor.html]

NamedColor


---


|  |  |  |
| --- | --- | --- |
| **Overview** \| **Package** \| **Class** \| **Tree** \| **Deprecated** \| **Index** \| **Help** \| **Weka's home** | |  |
| **PREV CLASS**   **NEXT CLASS** | **FRAMES**    **NO FRAMES**     **All Classes** |  |
| SUMMARY: NESTED \| FIELD \| CONSTR \| METHOD | DETAIL: FIELD \| CONSTR \| METHOD |  |


---


## weka.gui.treevisualizer Class NamedColor

```
java.lang.Object
  weka.gui.treevisualizer.NamedColor
```

---

``` public class NamedColor extends java.lang.Object ```

This class contains a color name and the rgb values of that color

**Version:**
:   $Revision: 7059 $

**Author:**
:   Malcolm Ware (mfw4@cs.waikato.ac.nz)

---

| **Field Summary** | |
| --- | --- |
| `java.awt.Color` | `m_col`             The actual color object |
| `java.lang.String` | `m_name`             The name of the color |


**Constructor Summary**

`NamedColor(java.lang.String n,
int r,
int g,
int b)`


**Method Summary**

**Methods inherited from class java.lang.Object**

`equals, getClass, hashCode, notify, notifyAll, toString, wait, wait, wait`

**Field Detail**

### m\_name

```
public java.lang.String m_name
```

:   The name of the color

---


### m\_col

```
public java.awt.Color m_col
```

:   The actual color object


**Constructor Detail**

### NamedColor

```
public NamedColor(java.lang.String n,
                  int r,
                  int g,
                  int b)
```

**Parameters:**: `n` - The name of the color.: `r` - The red component of the color.: `g` - The green component of the color.: `b` - The blue component of the color.


---


|  |  |  |
| --- | --- | --- |
| **Overview** \| **Package** \| **Class** \| **Tree** \| **Deprecated** \| **Index** \| **Help** \| **Weka's home** | |  |
| **PREV CLASS**   **NEXT CLASS** | **FRAMES**    **NO FRAMES**     **All Classes** |  |
| SUMMARY: NESTED \| FIELD \| CONSTR \| METHOD | DETAIL: FIELD \| CONSTR \| METHOD |  |


---
